# Frequent exacerbators of chronic obstructive pulmonary disease have distinguishable sputum microbiome signatures during clinical stability

**DOI:** 10.3389/fmicb.2022.1037037

**Published:** 2022-12-01

**Authors:** Xiaomin Dang, Yongyong Kang, Xiaojian Wang, Wen Cao, Minhui Li, Ying He, Xinjie Pan, Kai Ye, Dan Xu

**Affiliations:** ^1^Department of Respiratory and Critical Care Medicine, The First Affiliated Hospital of Xi’an Jiaotong University, Xi’an, China; ^2^Genome Institute, The First Affiliated Hospital of Xi’an Jiaotong University, Xi’an, China; ^3^Center for Mathematical Medical, The First Affiliated Hospital of Xi’an Jiaotong University, Xi’an, China; ^4^Chang’an District Hospital, The First Affiliated Hospital of Xi’an Jiaotong University, Xi’an, Shaanxi, China; ^5^Ministry of Education (MoE) Key Laboratory for Intelligent Networks and Network Security, Faculty of Electronic and Information Engineering, Xi’an Jiaotong University, Xi’an, China; ^6^Ministry of Education (MoE) Key Laboratory of Biomedical Information Engineering, School of Life Sciences and Technology, Xi’an Jiaotong University, Xi’an, China

**Keywords:** COPD, frequent exacerbation, sputum microbiome, *stenotrephomonas*, biomarker

## Abstract

**Introduction:**

Frequent exacerbation phenotype of chronic obstructive pulmonary disease (COPD) represents a more concerning disease subgroup requiring better prevention and intervention, of which airway microbiome provides new perspective for further exploration.

**Methods:**

To investigate whether frequent exacerbators of COPD have distinguishable sputum microbiome during clinical stability, COPD patients at high disease grades with or without frequent exacerbation were recruited for sputum microbiome analysis. Sputum samples were collected during clinical stability and underwent 16S rRNA sequencing, which was then subjected for amplicon sequence variants (ASVs)-based microbiome analysis.

**Results:**

Our results revealed that compared with healthy controls and infrequent exacerbators, frequent COPD exacerbators have distinguishably dysbiotic sputum microbiome, as featured by fewer ASVs features, lower alpha diversity, distinct beta diversity patterns. Further taxonomic compositional analysis illustrated the structural distinctions between frequent COPD exacerbators and infrequent exacerbators at differential taxa levels and highlighted *Stenotrephomonas* due to its prominent elevation in frequent COPD exacerbators, providing a promising candidate for further exploration of microbiome biomarker. Moreover, we also demonstrated that frequent exacerbation phenotype is distinguishable from infrequent exacerbation phenotype with respect of functional implications.

**Conclusion:**

Our study demonstrated the first positive correlation between the frequent exacerbation phenotype of COPD and the sputum microbiome during clinical stability in a single-center Chinese COPD cohort and provide potential diagnostic and therapeutic targets for further investigation.

## Introduction

Chronic obstructive pulmonary disease, which is characterized by irreversible airflow obstruction and unresolved inflammation, poses a significant threat to the health and life quality of people (Rabe and Watz, 2017). Acute exacerbations of COPD (AECOPD) are the major drivers in the clinical course of COPD since each exacerbation would cause a rapid loss of lung function that is partially unrecoverable and thus deteriorate the situation in the long term (Anzueto, 2010; Hurst et al., 2010). The frequent exacerbator phenotype (defined as the occurrence of ≥ 2 COPD acute exacerbations within 1 year) is of particular significance and is now recognized as a distinct clinical subgroup (Donaldson et al., 2002; Wedzicha and Seemungal, 2007), which is associated with poorer clinical outcomes and occurs stably across disease severities. These frequent exacerbators, consisting of 22% of the COPD population (Wilkinson et al., 2019), are more likely to have increased hospital admissions, comorbidities, and mortality, and are therefore a priority for research and treatment (Mirza et al., 2018). The current practical way to predict the frequent exacerbator phenotype was a history of exacerbation in the previous year, lacking better prognostic measurement due to a limited understanding of the etiology of frequent exacerbation (Anzueto, 2010; Halpin et al., 2017; Ouaalaya et al., 2020). A better understanding of this concerning phenotype and exploration of a novel biomarker for better diagnosis is crucial for developing precision-medicine strategies.

The alteration of the airway microbiome in course of COPD has been well-recognized and extensively documented (Dickson et al., 2014; Segal et al., 2014; Mayhew et al., 2018; Toraldo and Conte, 2019). There is compelling evidence that in comparison with a healthy population, patients with COPD, nevertheless during clinical stability or exacerbation, have distinct microbiota inhabiting respiratory and gastroenteric tracts, with a significant shift between different disease statuses (Sze et al., 2012; Wang et al., 2016; Wang Z. et al., 2018; Bowerman et al., 2020; Leiten et al., 2020), implying the potential involvement of microbiome in the progression of COPD. Of particular interest, the occurrence of AECOPD, which is mostly triggered by infection, is usually accompanied by a significant compositional and functional shift of airway microbiome (Molyneaux et al., 2013), as evidenced by an increase of Proteobacteria, especially attributable to the elevation of *Haemophilus* and *Moraxella* during exacerbations (Sethi et al., 2002; Dickson et al., 2014; Wang Z. et al., 2018). It is also well-perceived that even in the stable phase of COPD, the airway microbiome exhibits distinct features between different inflammatory endotypes, and more intriguingly, shows remarkable association with mortality of the patients (Leitao Filho et al., 2019; Wang et al., 2020b; Dicker et al., 2021). Moreover, emerging studies began to notice that upper airway (sputum) microbiome composition at clinical stability is correlated with the exacerbation frequency status, despite the inconsistency from different cohorts (Yang et al., 2021). One possible explanation is that the frequency of exacerbation events experienced by an individual may contribute to the destabilization of the lung microbiome, such that frequent exacerbators may be associated with greater dysbiosis than infrequent exacerbators. Nonetheless, conclusions from different cohorts were inconsistent on whether the frequent exacerbators have distinct sputum microbiomes during clinical stability. Data from the COPDMAP cohort revealed the first significant correlation of alpha and beta diversities with a historical number of exacerbations per year, in spite of remarkable variations among the sub-cohorts at three different cities (Wang Z. et al., 2018). The latter AERIS cohort study further demonstrated that the sputum microbiome became more distinct with greater exacerbation frequency, with *Moraxella* being the highest positive correlated genus (Mayhew et al., 2018; Wilkinson et al., 2019). A case-control observational study carried out on 22 patients with COPD who were recruited at the Minneapolis VA Medical Center in the US showed that upper airway microbiota alpha diversity index by Shannon entropy is significantly lower in frequent COPD exacerbators than infrequent exacerbators (Pragman et al., 2019). On the contrary, a recent study on the east Scottish COPD cohort did not detect a clear distinct clustering of sputum microbiome based on exacerbation frequency, although differences at both phylum and genus levels were observed (Dicker et al., 2021). Moreover, two studies on small-number of Chinese cohorts demonstrated that there were no significant community shifts between the patients that were frequent and infrequent exacerbators (Wang et al., 2020a), and the species evenness according to the Shannon index was similar in the low-risk vs. high-risk exacerbation groups (Yang et al., 2021). Although it is hypothesized that the airway microbiome is differentially associated with the exacerbation status, discrepancies from previous studies as mentioned above necessitate a further investigation of the detailed signature of airway microbiome in frequent COPD exacerbators in comparison with infrequent exacerbation cohorts.

It is also noted that most of these previous COPD airway microbiome studies were carried out on Caucasian cohorts with only two recent reports on a smaller number of Chinese cohorts (containing 98 patients with COPD and 78 patients with COPD, respectively) (Wang et al., 2020a; Yang et al., 2021). On the other hand, China has the largest population of patients with COPD with an estimation of 100 million, consisting of 33% of the global cases, which is still undergoing fast growth (Vestbo and Mathioudakis, 2018; Wang C. et al., 2018). More importantly, it has also been realized that Chinese COPD cohorts have their own special features due to their unique genetic, environmental, and medication background, reinforcing the necessity to deepen the understanding of the COPD airway microbiome in stratified Chinese cohorts (Wang et al., 2013; Fang et al., 2018; Vestbo and Mathioudakis, 2018).

**FIGURE 1:**
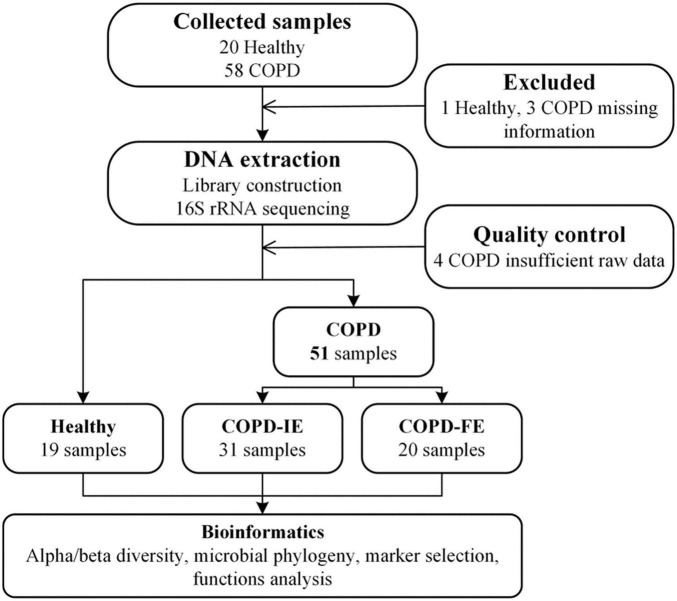
Study design and flow diagram (Mirzayi et al., 2021). A total of 78 subjects were included. After exclusion procedures and data quality controls, 19 healthy samples, 31 COPD-IE samples, and 20 COPD-FE samples were included for bioinformatic analysis. COPD, Chronic obstructive pulmonary disease; COPD-IE, COPD with infrequent exacerbations; COPD-FE, COPD with frequent exacerbations.

To investigate whether frequent exacerbators in the Chinese COPD cohort have distinguishable sputum microbiome during clinical stability, 78 volunteers (including 58 patients with COPD and 20 matched healthy controls) were recruited in the Shaanxi province of northwest China and stratified into a healthy control group, frequent COPD exacerbators, and infrequent exacerbators according to COPD exacerbation status in the past year (Figure 1). Sputum during clinical stability was collected and processed for 16S rRNA sequencing. Our analysis revealed that compared with healthy controls and infrequent exacerbators, frequent COPD exacerbators have distinct sputum microbiomes, as featured by lower alpha diversity, distinct beta diversity patterns, unique compositional structure, and distinct putative functions. Our study demonstrated the first positive correlation between the sputum microbiome during clinical stability and the frequent exacerbation phenotype in the Chinese COPD cohort and provided potential diagnostic and therapeutic targets for further investigation.

## Materials and methods

### Volunteer recruitment, and inclusion and exclusion criteria

In total, 58 patients with severe COPD and 20 matched healthy volunteers were recruited in the First Affiliated Hospital of Xian Jiaotong University and Changan Hospital of Shaanxi Province with the approval of the ethics committee of the hospitals and consent from all the patients and healthy volunteers. All donors read and signed the consent form before sample collection. All patients with COPD met the diagnostic criteria of severe and very severe COPD according to GOLD guidelines (GOLD stages III-IV) and were also assessed for exacerbation frequency. GOLD classification for disease severity was based on a pulmonary function test. For patients with COPD, the inclusion criteria were as follows: (1) confirmed diagnosis of severe and very severe COPD according to the GOLD guidelines (post-bronchodilator forced expiratory volume in 1s FEV_1_% < 50%) and (2) routine inhaled corticosteroids (ICS) medication. The exclusion criteria were as follows: (1) COPD exacerbation or use of antibiotics in the past 1 month; (2) asthma diagnosis; (3) malignancy; (4) diagnosis of autoimmune diseases; and (5) use of supplemental oxygen.

### Sputum sample collection and DNA extraction

Spontaneous or induced sputum was collected after having the subjects swish sterile water in their mouths and placed in DNA-free Petri dishes. Sputum plugs, which contained the most viscous material, were then picked up and isolated from saliva. All samples were weighed and frozen at −80°C until sample processing. Negative control samples consisted of unused sterile water and DNA contamination of reagents and equipment was evaluated using extraction controls, which were processed and analyzed alongside the experimental samples.

After thawing of the sputum samples, total bacterial genomic DNA samples were extracted using the Fast DNA SPIN extraction kits (MP Biomedicals, Santa Ana, CA, USA), following the instructions of the manufacturer. The quantity and quality of extracted DNA were measured using a NanoDrop ND-1000 spectrophotometer and agarose gel electrophoresis, respectively.

### 16S rRNA gene sequencing

PCR amplification of the bacterial 16S rRNA gene V3–V4 region was performed. In brief, sample-specific barcodes were incorporated into the primers for multiplex sequencing. 16S rRNA V3-V4 amplicons were generated *via* 20 PCR amplification cycles using primers 5′-ACTCCTACGGGAGGCAGCA-3′ and 5′-GGACTACHVGGGTWTCTAAT-3′. Following purification and quantification, amplicons were pooled in equal amounts, and pair-end 2 × 250 bp sequencing was performed using the NovaSeq-PE250 platform with NovaSeq Reagent Kit at the Shanghai Personal Biotechnology Co., Ltd., (Shanghai, China).

### Amplicon sequence variants clustering and taxonomy annotation

All raw reads were processed by QIIME2 (Bolyen et al., 2019) (q2cli version 2021.8.0) pipeline through the following steps: (a) raw sequencing data produced as QIIME 2 artifacts; (b) used command “qiime dada2 denoise-paired” to perform quality control and “qiime feature-table” to filter the samples, features, and sequences (Callahan et al., 2016). All ASV features need to occur in at least two samples. (c) The features were annotated with the command “qiime feature-classifier classify-sklearn” based SILVA database (138.1-ssu-nr99) that was trained by “qiime feature-classfier fit-classifier-naive-bayes” (extracted V3 and V4 regions from full-length sequences). In the final annotation file, labeled as archaea and uncultured taxon were removed.

### Bacterial diversity and taxonomic analysis

By a sampling-based ASVs analysis (the sampling depth is 34,407, the minimum number of sequences in all samples), bacterial diversity was determined and shown by the Shannon index, the ACE index, and the Simpson index, which used a series of commands with “qiime diversity.” PCoA was conducted by QIIME2 plugins to display the microbiome space between both the group samples.

Bacterial taxonomic analyses and comparisons between both groups by the Wilcoxon rank-sum test were conducted at the genus level. Based on the normalized relative abundance matrix, the LEfSe (Segata et al., 2011) method was applied to analyze sputum microbial characterization between COPD (COPD-IE or COPD-FE) and healthy controls. This method first used the Kruskal–Wallis rank-sum test (default *p* < 0.05) to detect features with a significant differential abundance, then evaluated the effect size of each feature by LDA [LDA score (log10) = 2 as default cut-off value].

### Functional annotation of 16S rRNA gene based on the Kyoto encyclopedia of genes and genomes profile

The Enzyme Commission (EC) numbers, KEGG orthologs (KOs), and the KEGG pathway/module profile were constructed by the PICRUSt2 (Douglas et al., 2020) version 2.4.2, and 16S rRNA marker gene sequences were used to predict the microbial community function profiles. PICRUSt2, the algorithm which contains a large up-to-date database of gene families and reference genomes, was employed to annotate denoised ASVs features with predicted functions based on alignment against EC numbers and KOs. All the shuffled ASV features are placed into a reference tree, which is used as the basis of functional predictions and subsequent pathways enrichment analysis.

### Statistical analysis

Differences between subjects in COPD and healthy controls were compared by Wilcoxon rank-sum test for non-normal continuous variables. Statistical analyses were performed using the R stats package. Statistical significance was defined by *p* < 0.05 (two-sided). Predicted functional differences were performed by STAMP (Parks et al., 2014) software (version 2.1.3) with Welch’s *t*-test (*p* < 0.01).

## Results

### Characteristics of the study participants

Only severe-to-very severe (GOLD stages III and IV with FEV1% < 50%) COPD patients with routine inhaled corticosteroids (ICS) and long-acting bronchodilator treatment were included in this study to eliminate the influence of differential severity of COPD, FEV1%, and medication, which was reported to be correlated with the airway microbiome. No differences were observed between the three groups on age, smoking history, FEV1%, PEF, and COPD severity. Clinical information of the participants is presented in Table 1. All the COPD sputum samples were collected during clinical stability with no acute exacerbation event and antibiotic usage for at least 1 month. Frequent exacerbation phenotype was defined as ≥ 2 exacerbations in the past 12 months. A total of 78 samples from different types of volunteers were collected prospectively. After rigorous diagnosis and exclusion procedures, eight samples were excluded from the final analysis due to missing information or lack of sufficient quality and quantity of raw data. Therefore, 19 healthy samples, 31 infrequent COPD exacerbators (COPD-IE) samples, and 20 frequent COPD exacerbators (COPD-FE) samples were included for the following bioinformatic analysis (Figure 1).

**TABLE 1:** Characteristics of the study population.

	Infrequent exacerbation (*n* = 31)	Frequent exacerbation (*n* = 20)	Healthy control (*n* = 19)
Gender, n (M/F)	22/9	17/3	10/9
Age	67 (60, 72.5)	67 (57.75, 73.25)	62 (57, 68)
BMI (kg/m^2^)	20.76 (18.70, 24.16)	21.96 (19.81, 23.38)	25.26 (23.05, 27.68)
Current/ex-smokers	17	17	7
FVC	1.89 (1.48, 2.36)	2.2 (1.61, 2.37)	3.05 (2.14, 3.91)
FEV1	0.86 (0.70, 1.07)	0,88 (0.69, 1.24)	2.32 (1.68, 2.93)
FEV1%pre	34.17 (26.17, 41.58)	35.04 (24.07, 43.18)	94.9 (86.2, 110.07)
FEV1/FVCratio	45.32 (39.55, 51.47)	42.36 (37.94, 48.40)	77.14 (74.99, 79.83)
FEV1/FVCratio%pre	54.04 (47.10, 61.09)	50.63 (45.35, 57.72)	92.40 (86.25, 94.83)
PEF	2.16 (1.52, 2.56)	2.33 (2.00, 2.56)	4.73 (4.43, 7.85)
PEF%pre	27.63 (24.40, 33.79)	28.66 (25.78, 37.65)	89.70 (74.82, 97.16)
**GOLD stage**			
III	21	11	—
IV	10	9	—
Blood erythrocyte (×10^12^/L)	4.50 (4.11, 4.72)	4.8 (4.40, 5.02)	4.38 (4.14, 4.86)
Hemoglobin (g/L)	143 (127, 149)	151 (135.5, 157.25)	141.50 (133.50, 155)
blood platelet (×10^9^/L)	227 (202, 305.5)	202.5 (158.5, 237.5)	190 (182.75, 211.75)
hs-CRP (10 mg/L)	6.4 (1.96, 18.24)	4.06 (1.94, 14.43)	—
Blood white cells (×10^9^/L)	7.07 (6.26, 7.69)	6.61 (4.88, 7.94)	5.83 (5.26, 6.24)
Blood neutrophils (%)	67 (62.75, 74.85)	62.7 (56.08, 74.65)	59.90 (57.55, 68.05)
Blood eosinophils (%)	1.90 (0.75, 3.05)	4.4 (1.95, 5.2)	1.95 (1.35, 2.55)
Blood lymphocytes (%)	21.90 (18.20, 29.15)	25.7 (16.7, 29.5)	28.80 (23.13, 34.28)
Blood monocytes (%)	6.30 (5.05, 7.65)	5.75 (5.08, 7.4)	6.05 (4.70, 6.75)

Data are presented as IQR, BD, bronchodilator; FEV1, forced expiratory volume in 1s.

### Frequent exacerbation sputum microbiome has the lowest number of observed amplicon sequence variants features

**FIGURE 2:**
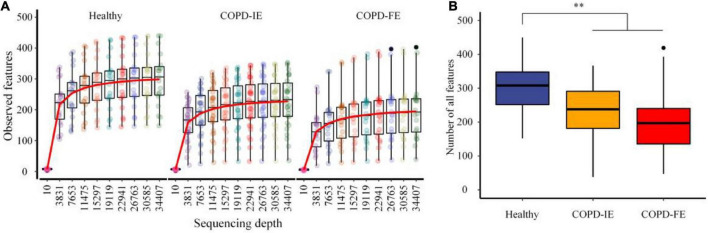
The rarefaction analysis between the number of samples and the number of amplicon sequence variants (ASVs) features. **(A)** Sampling depth and ASVs features. **(B)** All observed ASVs features in different groups. COPD, Chronic obstructive pulmonary disease; COPD-IE, COPD with infrequent exacerbations; COPD-FE, COPD with frequent exacerbations. ^**^p < 0.01.

After quality control, 2,363 amplicon sequence variants (ASVs) features that occurred in at least two samples were obtained. The mean frequency per sample is 67,912, with 34,407 being the lowest. When the sequencing depth reaches the lowest frequency, the ASVs features of all samples reach the plateau, guaranteeing sufficient data quality for further analysis (Figure 2A and Supplementary Figure 1). While there are 306 ASVs features on average in the healthy control group, there are significantly fewer 221 ASVs features in COPD groups (*p* = 0.001596), with frequent COPD exacerbation sputum microbiome having the smallest number of all the observed ASVs features (mean counts = 202) (Figure 2B and Supplementary Figure 1), indicating the underlying differences waiting for further elucidation.

### Frequent exacerbation sputum microbiome has a lower alpha diversity

**FIGURE 3:**
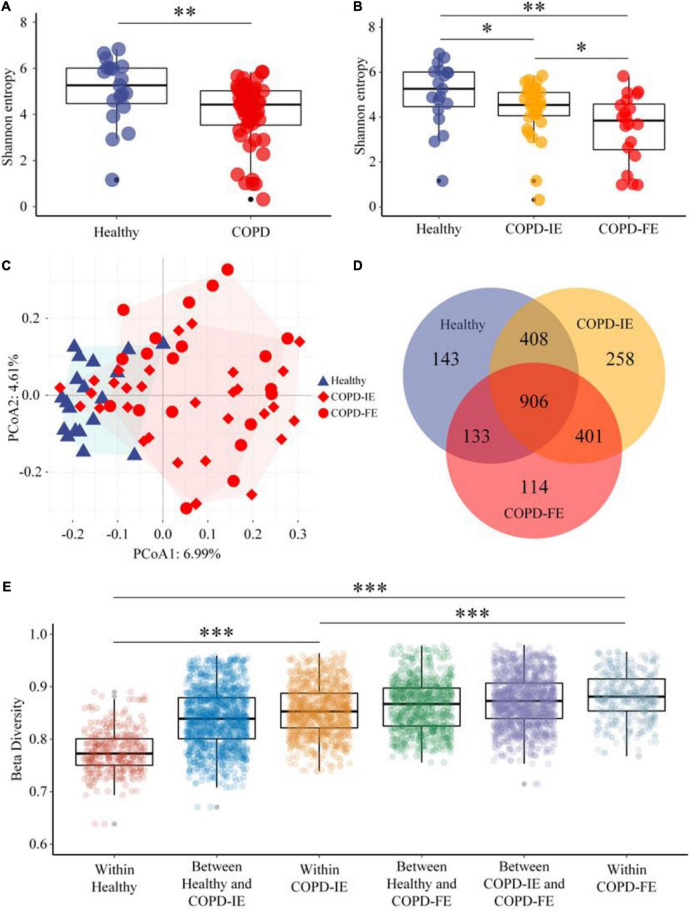
Sputum microbiome diversity analysis. **(A)** As estimated by Shannon entropy, sputum microbial diversity was significantly decreased in chronic obstructive pulmonary disease (COPD) (*n* = 51) compared with that in the healthy controls (*n* = 19). **(B)** Shannon entropy of COPD-IE and COPD-FE group in comparison with healthy controls. **(C)** PCoA analysis based on amplicon sequence variants (ASVs) features distribution showed that healthy subjects form a distinguishable cluster differing from COPD groups while a large overlap of clustering was detected between frequent and infrequent exacerbation phenotypes. **(D)** Venn diagram displaying the overlaps between groups. **(E)** Beta diversity analysis based on the Jaccard distance and Wilcoxon test demonstrated that besides the expected significant differences between the healthy control group and COPD sub-cohorts (COPD-IE and COPD-FE), the distinction between COPD-IE and COPD-FE was also statistically significant. *p < 0.05; ^**^p < 0.01; ^***^p < 0.001.

Alpha diversity, which reflects the stability and robustness of an ecosystem, was then examined to illustrate the differences between the groups. In consistence with previous studies, COPD subjects in our study, regardless of the exacerbation frequency, exhibited lower alpha diversity compared with healthy controls as measured by Shannon entropy (*p* = 0.0045), ACE index (*p* = 0.0017), and Simpson index (*p* = 0.0324) (Figure 3A and Supplementary Figure 2). Moreover, Shannon entropy, the major alpha diversity parameter which takes both richness and evenness into account, further distinguished frequent and infrequent phenotypes of COPD subjects by a significant drop in the COPD-FE group (*p* = 0.0306), suggesting an unneglectable correlation between frequent exacerbation and more dysbiotic sputum microbiome (Figure 3B).

### Beta diversity of sputum microbiome distinguishes frequent exacerbation phenotype

We next attempted to answer the question of whether frequent exacerbation correlates with distinct sputum microbiome, which is also the most controversial issue in the previous studies. To understand the degree of dissimilarity between the different groups, beta diversity was employed to determine the extent of microbial ecosystem differentiation between the groups. PCoA clustering analysis based on ASVs features indicated that samples from healthy subjects form a distinguishable cluster differing from COPD groups while a large overlap of clustering was detected between frequent and infrequent exacerbation phenotypes (Figure 3C). Nonetheless, all-feature-derived Venn diagram showed that besides the large portion of shared features, each group possessed its own unique features (Figure 3D). Beta diversity analysis based on Jaccard distance and Wilcoxon test that measures the similarity in bacterial composition between groups demonstrated that besides the expected significant differences between the healthy control group and COPD cohort as a whole or as two sub-cohorts (COPD-IE and COPD-FE) (*p* < 0.001), the distinction between COPD-IE and COPD-FE was also statistically significant (*p* < 0.001) (Figure 3E and Supplementary Figure 2), implicating a different airway microbial assemblage between these two subgroups based on exacerbation frequency differentiation. Further characterization of the difference would provide new perspectives to explore unique features of frequent exacerbation phenotype.

### Taxonomic composition and alterations of the sputum microbiome in different chronic obstructive pulmonary disease phenotypes

**FIGURE 4:**
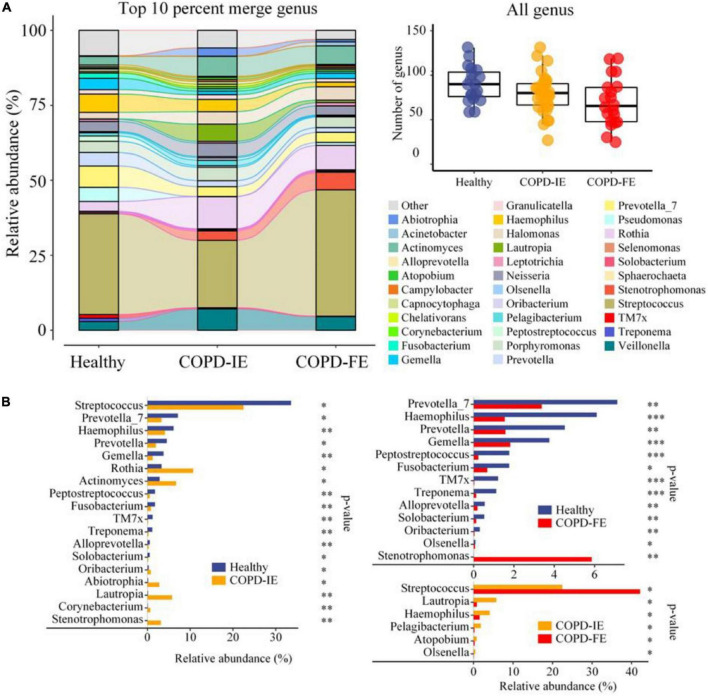
Phylogenetic profiles of the sputum microbiome in healthy controls and chronic obstructive pulmonary disease (COPD) groups (COPD-IE and COPD-FE). **(A)** Average compositions and relative abundance of the bacterial community in each group at the genus level (top 10% in each group). The inset shows a box plot summarizing the distributions of the numbers of all genera detected for different groups. **(B)** Differentially distributed genera between groups. *p < 0.05; ^**^p < 0.01; ^***^p < 0.001.

**FIGURE 5:**
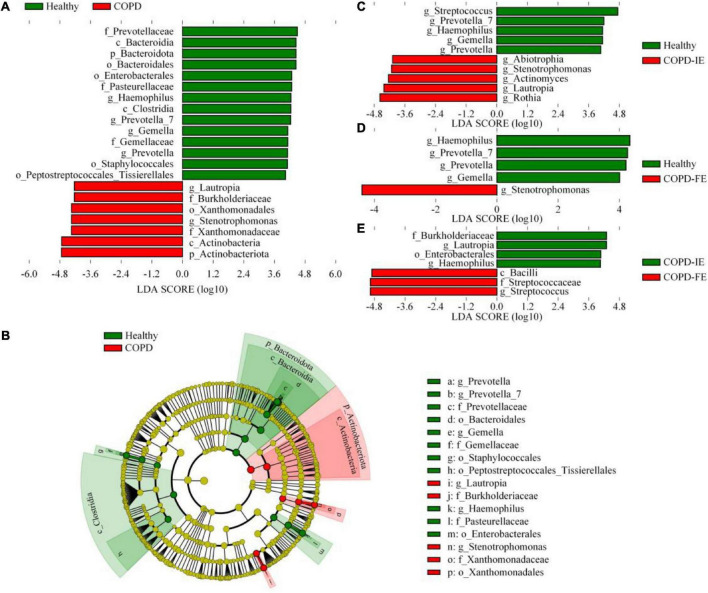
The LEfSe test of sputum microbiome [LDA score (log10) = 4]. **(A)** Differentially distributed microbial taxa of all ranks between chronic obstructive pulmonary disease (COPD) subjects and healthy controls. **(B)** The phylogenetic profile of the specific differentially distributed taxa. **(C–E)** Differentially distributed genera between groups. Statistical significance was defined by *p* < 0.05.

To unravel the details of the differences as detected in the diversity analysis above, taxonomic composition and relative alterations between the groups were examined after all the ASVs features were annotated to a hierarchy of differential taxa ranks. At the genus level, few taxa in each sample (70, 58–82, 95% confidence) were detected in the COPD-FE group compared with healthy control (90, 81–99, 95% confidence) and COPD-IE (80, 72–87, 95% confidence) groups (Figure 4A). Top 10% of the genus in each group were further compared to illustrate the alterations of the most abundant genus between the groups, as visualized in Sankey diagram in Figure 5A. We then, logically, examined the specific genera of each group that is responsible for group differentiation. Compared with healthy controls, genera increased in abundance in COPD-IE including *Actinomyces*, *Rothia*, *Lautropia*, *Stenotrophomonas, Abiotrophia*, and *Oribacterium*. While genera decreased in COPD-IE including *Streptococcus*, *Prevetella*, *Prevetella-7*, *Gemella*, *Haemophilus*, *Peptostreptococcus*, *Fusobacterium*, *TM7x*, *Treponema*, *Alloprevetella*, and *Solobacterium*. Intriguingly, when comparing healthy controls with COPD-FE, most of the significantly different genera were decreased in COPD-FE samples, with *Stenotrophomonas* being the only one with a remarked 837-fold elevation in abundance. Additionally, a comparison between COPD-FE and COPD-IE samples revealed that there were six genera differing these two groups, with *Streptococcus* increased in COPD-FE and five other genera including *Lautropia*, *Haemophilus*, *Pelagibacterium*, *Atopobium*, and *Olsenella* decreased (Figure 4B).

### Sputum microbiome taxonomic indicators of frequent exacerbation phenotype

To further identify sputum microbiome taxa contributing to the distinction between frequent exacerbation and infrequent exacerbation phenotypes, the LEfSe test, which takes both differences in abundances and frequency into account, was employed on all the taxa for searching potential biomarkers. Comparison of abundances by Kruskal–Wallis test with linear discriminant analysis (LDA) selection [LDA score (log10) = 4 as cutoff value (a higher cutoff value of 4 rather than 2 was chosen to get a better distinction between different groups)] revealed that differences between healthy controls and patients with COPD were detected at differential levels of taxa as reported in previous studies. At the bacterial phylum level, Actinobacteriota was enriched in COPD while Bacteroidota was depleted; this is consistence with the analysis at the class level showing that *Actinobacteria* and *Bacteroidia* were, respectively, enriched in the COPD group and healthy group (Figures 5A,B). At the order level, Xanthomonadales was elevated in the COPD group with decreased Bacteroidales, Enterobacterales, Staphylococcales, and Peptostreptococcales. At the family level, Burkholeriaceae and Xanthomonadaceae were enriched in COPD with Prevotellaceae, Pasteurellaceae, and Gemellaceae depletion. The most prominent genera in the COPD group were *Stenotrephomonas* and *Lautrophia* while *Haemophilus*, *Prevotella*, and *Prevetella-7* were enriched in healthy controls. At the genus level, compared with healthy and COPD-IE or COPD-FE, *Stenotrephomonas* was enriched in both groups (Figures 5C,D). Comparatively, the dissimilarity between COPD-IE and COPD-FE was less pronounced since fewer different features were observed when using the same cutoff value with an LDA score (log10) = 4. The highest rank of distinguishable taxa between the two subgroups is the Bacilli class which was elevated in COPD-FE, in addition to Streptococcaceae at the family level and *Streptococcus* at the genus level. Among the depleted taxa in COPD-IE, Burkholderiaceae at the family level, Enterobacterales at the order level, and *Lautropia* and *Haemophilus* at the genus level were identified, as demonstrated in Figure 5A. Overall, our results confirmed that in addition to the well-documented significant compositional shifts from healthy to COPD, there were also distinct compositional features of sputum microbiome between COPD-IE and COPD-FE phenotypes (Figure 5E and Supplementary Figure 3).

**FIGURE 6:**
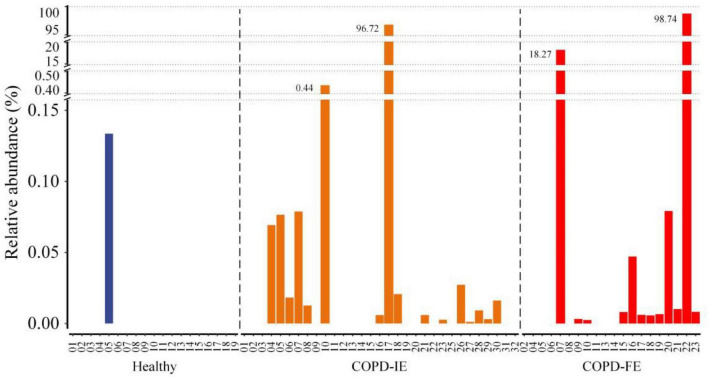
The frequency and relative abundance of the *Stenotrephomonas* in healthy controls, chronic obstructive pulmonary disease with infrequent exacerbations (COPD-IE), and COPD with frequent exacerbations (COPD-FE).

Among the differentially distributed taxa between different COPD subgroups, *Stenotrephomonas* was of particular interest due to its prominent increase in COPD-FE subjects. Closer examination of its abundance revealed that this genus barely existed in the healthy samples (1/19) with quite a low average relative abundance (0.007), while it was found in a significantly larger proportion of patients with COPD (COPD-IE: 16/31; COPD-FE: 12/20). The mean relative abundance analysis implicated that frequent exacerbation phenotype was associated with a higher abundance of this opportunistic pathogenic genus (5.86% in COPD-FE and 3.15% in COPD-IE) (Figure 6), providing a promising candidate for further exploration of microbiome biomarkers.

### Functional implications of the altered sputum microbiome in chronic obstructive pulmonary disease subjects

**FIGURE 7:**
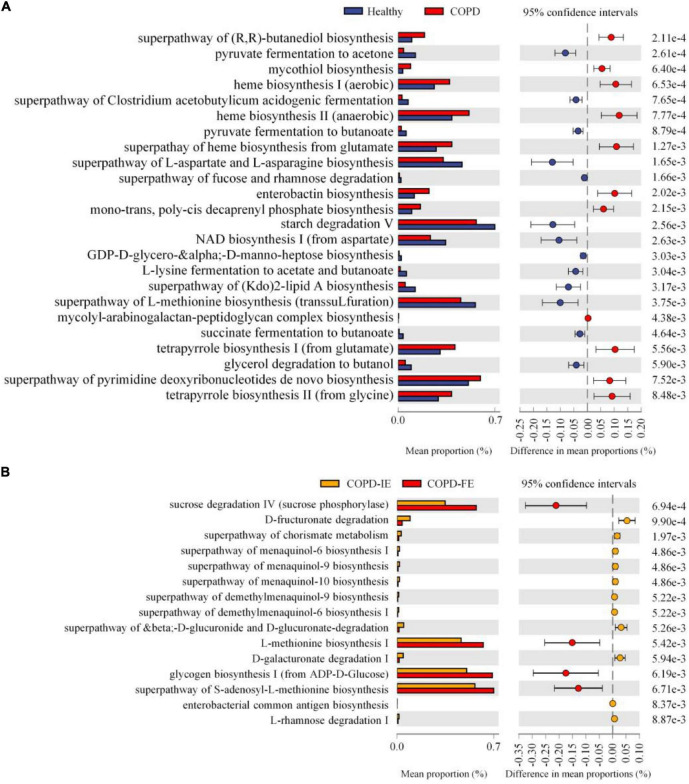
Function prediction of sputum microbiome by PICRUSt2. Based on predicted EC numbers and pathways, **(A)** 11 predicted microbial functions were remarkably increased, while 13 functions were remarkably decreased in chronic obstructive pulmonary disease (COPD) compared with healthy controls. **(B)** Four predicted microbial functions were remarkably increased, while 11 functions were remarkably decreased in COPD with frequent exacerbations (COPD-FE) compared with COPD with infrequent exacerbations (COPD-IE). [Welch’s *t*-test (two-sided): *p* < 0.01].

The KEGG orthologs (KOs), EC numbers, and MetaCyc/KEGG pathway profile were constructed using the PICRUSt2 pipeline, and the 16S rRNA marker gene sequences were used to predict the microbial community function profiles. Our results demonstrated that compared with healthy controls, 24 pathways were significantly different in the COPD sputum microbiome, with 11 elevated and 13 downregulated in COPD samples (Figure 7A) (*p* < 0.01). Besides, the biosynthesis of enterobactin, a high-affinity siderophore that acquires iron for microbial systems (Raymond et al., 2003), was significantly promoted in COPD samples. Moreover, using the same threshold for enrichment analysis (*p* < 0.01), 15 pathways were found to differ between COPD-IE and COPD-FE groups. Two out of the four upregulated pathways in COPD-FE were related to glycometabolism, including sucrose degradation and glycogen biosynthesis, with the other two related to L-methionine biosynthesis (Figure 7B).

To further elucidate the functional difference between COPD-IE and COPD-FE sputum microbiome, KEGG orthologs (KOs) were performed to explore the differential pathways between the two groups, which found out that besides the elevated glycometabolism pathways as revealed in the EC analysis, two inflammation and infection pathways were also increased in COPD-FE samples, including the inflammasome-activating NOD-like-receptor-signaling pathway (Franchi et al., 2009) and *Staphylococcus aureus* infection pathway, coinciding with the adverse clinical outcomes of frequent exacerbation phenotype (Supplementary Figure 4).

## Discussion

We present the first analysis to focus on exploring the distinguishability of upper airway microbiome between frequent and infrequent exacerbation phenotypes of patients with COPD during their stability phase in the Chinese cohort to complement previous work focused on the inflammatory endotypes. Frequent exacerbation poses a significant threat to the life quality and life expectancy of patients with COPD and thus represents a distinct disease subgroup that requires better diagnostic and therapeutic means for effective prevention of frequent exacerbation and control of the devastating symptoms (Hurst et al., 2010). The current way to predict the frequent exacerbation phenotype is mainly based on the exacerbation history over the past year, lacking accurate prognostic biomarkers to discriminate the phenotype for better and earlier interventions (Soriano et al., 2015). Microbial communities residing in human gastrointestinal and respiratory tracts have been reported to differ between patients with COPD and healthy controls, providing a new perspective to explore potential biomarkers to further distinguish subgroups of patients with COPD, especially the frequent exacerbation phenotype (Segal et al., 2014; Bowerman et al., 2020). Since most of the exacerbation events are caused by microbial infections, it is attempting to hypothesize that the airway microbiome is differentially associated with the exacerbation status. However, several previous studies that attempted to validate this hypothesis have obtained inconsistent even contradicting conclusions on whether or not the sputum microbiome of frequent COPD exacerbators has distinct characteristics in composition and function in comparison with infrequent exacerbators. Three of the six related studies supported the notion that sputum microbiome features of frequent exacerbators are distinct from infrequent exacerbators, while opposite conclusions were drawn from the other three cohorts’ analyses (Pragman et al., 2019; Wilkinson et al., 2019; Wang et al., 2020a,b; Dicker et al., 2021; Yang et al., 2021). One of the major reasons for these discrepancies is the differential stratification of the patients. The severity of the disease, FEV1%, and medication status have been reported to influence the sputum microbiome compositional features (Garcia-Nuñez et al., 2014; Pragman et al., 2015; Contoli et al., 2017), as exampled by a significant shift toward increasing Proteobacteria with increasing COPD severity observed in the AERIS study (Wilkinson et al., 2019). To eliminate the influence of these recognized variables on the airway microbiome, this study exclusively recruited severe patients with COPD of GOLD stages III and IV with FEV1% of < 50% and routine ICS and bronchodilator medication, which were then further stratified into frequent exacerbators and infrequent exacerbators according to the exacerbation history in the last year. Moreover, due to the remarkable fluctuation of the sputum microbiome during exacerbation and antibiotics for treatment as reported previously, we only collected sputum samples during clinical stability that lasts at least 1 month to diminish the perturbation caused by antibiotics usage, which is also meant to explore potential biomarker during clinical stability phase to predict underlying frequent exacerbation phenotype.

Although emerging metagenomic sequencing technique provides remarkably more details for microbiome analysis (Bowerman et al., 2020; Wang et al., 2020a), 16S rRNA sequencing still represents an essential way to analyze clinical samples due to its less computational requirements and lower expense, which may facilitate the potential application in the clinical setting for diagnostic purposes. Nonetheless, better analysis methodology is required to get a finer resolution of the amplicon sequences since the historically dominated operational taxonomic units (OTUs)-based methods tend to sacrifice the subtle differences between the sequences due to its clustering nature based on similarity above a certain threshold (usually 97 or 99%). The recently developed denoising methods produced exact sequence variants or amplicon sequence variants (ASVs), instead of producing clusters to allow for a higher resolution than OTUs (Callahan et al., 2017). The benefits of the finer resolution brought by ASVs have been well-recognized in light of their obviously improved resolution and implied broad benefits that are derived from the status of ASVs as consistent labels with intrinsic biological meaning identified independently from a reference database (Wensel et al., 2022). Nevertheless, till now there have been only two studies employing ASVs-based pipelines to analyze the airway microbiome of patients with COPD, one of which is the first airway microbiome study on a Chinese cohort carried out by Wang et al. (2020a) recruited 98 patients with COPD from southeast China, showing that there were no significant community shifts between the patients with frequent and infrequent exacerbators (Wang et al., 2020a). In stark contrast, the other ASVs-based analysis on patients with COPD recruited at the Minneapolis VA Medical Center in the US unraveled that upper airway microbiota alpha diversity is significantly lower in frequent COPD exacerbators than infrequent exacerbators (Pragman et al., 2019). Deeper insight into the inconsistency between these two studies revealed that the ratio of severe patients was remarkably lower in Wang’s study (42 vs. 64%) with a commitment to higher predicted FEV1 (56.1 ± 2.7 vs. 41.16%) compared with the Minneapolis study, implicating the possibility that the difference would be pronounced in patients with higher grades of disease. This assumption was validated in our ASV-based study on severe patients with COPD with GOLD stage of III and IV with FEV1% of < 50%, which demonstrated that significant different features were found between frequent and infrequent exacerbation phenotypes.

Our first important result is that frequent exacerbators have more dysbiotic sputum microbiome, as evidenced by a dropped alpha diversity. Alpha diversity that measures the richness and evenness of the species in a given ecosystem is one of the most important parameters to evaluate the stability and robustness of the microbial communities, which was proven to decrease in the COPD-FE samples, consistent with the previous study on Minneapolis COPD cohort (Pragman et al., 2019) and studies on other chronic lung diseases such as cystic fibrosis, in which lower alpha diversity is associated with more severe diseases (Segal et al., 2014; Toraldo and Conte, 2019). We also noticed that in comparison with healthy controls, the decreased alpha diversity in patients with COPD reached statistical significance for all three indices including Shannon entropy, ACE, and Simpson, while the drop of alpha diversity of the COPD-FE group compared with COPD-IE group was statistically significant only in Shannon index but not in ACE and Simpson indices. Dropped alpha diversity usually indicates loss of symbiosis of a microbial ecosystem due to the unbalanced dominance of a few species, especially real and opportunistic pathogenic species introduced or outgrown in each infection-initiated exacerbation event (Koppen et al., 2015; Wang et al., 2016; Toraldo and Conte, 2019). The undermined alpha diversity in frequent exacerbation phenotype may be attributable to the repeated destabilization of the airway microbiome and therefore associated with greater dysbiosis than infrequent exacerbation phenotype, consistent with the general observation that lower alpha diversity is associated with worsening lung health.

More importantly, we also provide an affirmative answer to the controversial question of whether or not frequent exacerbators can be distinguished from infrequent exacerbators by unique sputum microbiome characteristics. Significant beta diversity differences based on Jaccard distance and Wilcox test analysis suggest that COPD-FE and COPD-IE sputum microbiome are dissimilar with respect to the compositional features, contradicting the studies on the Scottish cohort and Southeast China cohort but concordant with the conclusions from the COPDMAP study, AERIS study, and Pragman’s study on Minneapolis cohort (Wang Z. et al., 2018; Pragman et al., 2019). In the following identification of the specific taxa associated with frequent exacerbation phenotype, *Stenotrophomonas*, an emerging pathogenic genus, was selected to be the only elevated genera in the COPD-FE group. To our knowledge, it is the first time to establish a significant correlation between the frequent exacerbation phenotype and Stenotrophomonas genera elevation. The typical species of this genera, *Stenotrophomonas maltophilia*, is currently recognized as an emerging multidrug-resistant global opportunistic pathogenic genera, especially in immunocompromised patients and those with chronic diseases such as COPD presenting with signs and symptoms of infection (Brooke, 2012; Oladunjoye et al., 2020). Moreover, a comparison between COPD-FE and COPD-IE unraveled that Streptococcus-dominant profiles are associated with frequent exacerbation phenotype. It has been reported that patients with COPD with dominance due to *Streptococcus* had a high level of disease severity with impairment of quality of life and lung function impairment, and they had mortality equivalent to that of patients with *Haemophilus* dominance (Dicker et al., 2021).

Besides the compositional and taxonomic distinctions, we also demonstrated that the frequent exacerbation phenotype is distinguishable from the infrequent exacerbation phenotype with respect to functional implications as predicted by PICRUSt2 based on shuffled ASVs. Among the upregulated pathways, five tetrapyrrole and heme biosynthesis-related pathways were significantly promoted. It has been documented that excess free heme synthesis could cause oxidative damage and tissue inflammation, acting as a prototypic damage-associated molecular pattern (Dutra and Bozza, 2014), coinciding with the airway destruction and pronounced inflammation observed in patients with COPD. Similar to the taxonomic analysis, the functional distinctions are less pronounced between COPD-FE and COPD-IE subgroups than the comparison between healthy controls and COPD subjects. Nonetheless, there are still six pathways that were found to be elevated in frequent exacerbation phenotype, which consists of inflammasome-activating NOD-like-receptor-signaling pathway and *Staphylococcus aureus* infection pathway, indicating the higher risks of infection and inflammation which are believed to be the main drivers of frequent exacerbation.

The limitations of our study should be acknowledged. Our study was enrolled from the northwest China region, rendering the question of whether the discrepancy between Wang’s study on the southeast China cohort and our study is attributable to geography, which is still open to be answered. Multi-centered studies on large Chinese COPD cohorts are necessary to further address the current ambiguity. Although 16S ribosomal RNA sequencing is the most widely used method in the literature, its inherent limitations and biases undermined the fine resolution and interpretation of our results, necessitating further metagenomic sequencing and analysis to determine organism identity at the species level. Moreover, longitudinal profiling the patients with COPD reveals the dynamics of sputum alteration in frequent COPD exacerbators.

In conclusion, we showed that frequent exacerbators have distinguishable sputum microbiome characteristics during clinical stability in the Chinese COPD cohort. We identified taxonomic and functional differences between the two phenotypes, providing new possibilities for exploring diagnostic biomarkers to identify and predict underlying frequent exacerbators for better therapeutic prevention and intervention.

## Importance

Frequent exacerbation of COPD poses a significant threat to the life quality and life expectancy of patients and thus represents a distinct disease subgroup that requires better diagnostic and therapeutic means for effective prevention and intervention. The current way to predict the frequent exacerbation phenotype is mainly based on the exacerbation history over the past year, lacking accurate prognostic biomarkers for early discrimination of the phenotype. Although the airway microbiome provides a new perspective to explore potential biomarkers to identify COPD and its subtypes, discrepancies from previous studies necessitate a further investigation of the detailed signature of airway microbiome in frequent COPD exacerbators in comparison with infrequent exacerbation cohort for exploration of a novel specific biomarker for better diagnosis and therapeutics.

## Data availability statement

The clean sequence data reported in this article have been deposited in the Genome Sequence Archive in BIG Data Center (Wang et al., 2017, National Genomics Data Center Members and Partners, 2019), Chinese Academy of Sciences, under accession number: CRA006943 that are publicly accessible at https://ngdc.cncb.ac.cn/gsa.

## Ethics statement

The studies involving human participants were reviewed and approved by Ethics committee of The First affiliated Hospital of Xian Jiaotong University. The patients/participants provided their written informed consent to participate in this study.

## Author contributions

XD, KY, and DX conceived the idea and designed the study. KY and DX discussed and drafted the manuscript. YK and WC performed the data collection and analysis. XW, ML, YH, and XP established the COPD cohorts and collected the samples. All authors read and approved the final manuscript.

## Supplementary material

Supplementary Figure 1 The rarefaction analysis between the number of samples and the number of amplicon sequence variants (ASVs) features. **(A)** Sampling depth reaches the lowest frequency, ASVs features has reached the plateau. **(B)** All observed ASVs features in different groups.

Supplementary Figure 2 Sputum microbial diversity of patients with chronic obstructive pulmonary disease (COPD) was decreased. **(A)** As estimated by ACE index, Simpson index, sputum microbial diversity was significantly decreased in COPD (*n* = 51) compared with that in the healthy controls (*n* = 19). **(B)** Beta diversity analysis based on Jaccard distance and Wilcoxon test, distinction between healthy controls and COPD was statistically significant. **p* < 0.05; ^**^*p* < 0.01; ^***^*p* < 0.001. COPD, Chronic obstructive pulmonary disease.

Supplementary Figure 3 Crucial bacteria of sputum microbiome related to chronic obstructive pulmonary disease (COPD)-FE. The phylogenetic profile of the specific bacterial taxa and the major bacteria associated with COPD-IE and COPD-FE.

Supplementary Figure 4 Predicted functions related to frequent exacerbation phenotype. Based on the predicted KEGG orthologs (Kos) and Kyoto encyclopedia of genes and genomes (KEGG) pathways, 9 predicted microbial functions were remarkably increased, while 11 functions were remarkably decreased in COPD-FE compared with COPD-IE [LDA score (log10) = 2 is cutoff value]: *p* < 0.05. COPD, Chronic obstructive pulmonary disease; COPD-IE, COPD with infrequent exacerbations; COPD-FE, COPD with frequent exacerbations; LDA, linear discriminant analysis.
