# Supplementary material for: Frequent exacerbators of chronic obstructive pulmonary disease have distinguishable sputum microbiome signatures during clinical stability
Source: Front Microbiol. 2022 Dec 1;13:1037037. doi: 10.3389/fmicb.2022.1037037 (PMC9753979; doi:10.3389/fmicb.2022.1037037)
Supplement: Supplementary Figure 1 — The rarefaction analysis between the number of samples and the number of amplicon sequence variants (ASVs) features. (A) Sampling depth reaches the lowest frequency, ASVs features has reached the plateau. (B) All observed ASVs features in different groups. [file Data_Sheet_1.docx]

SUPPLEMENTAL MATERIAL


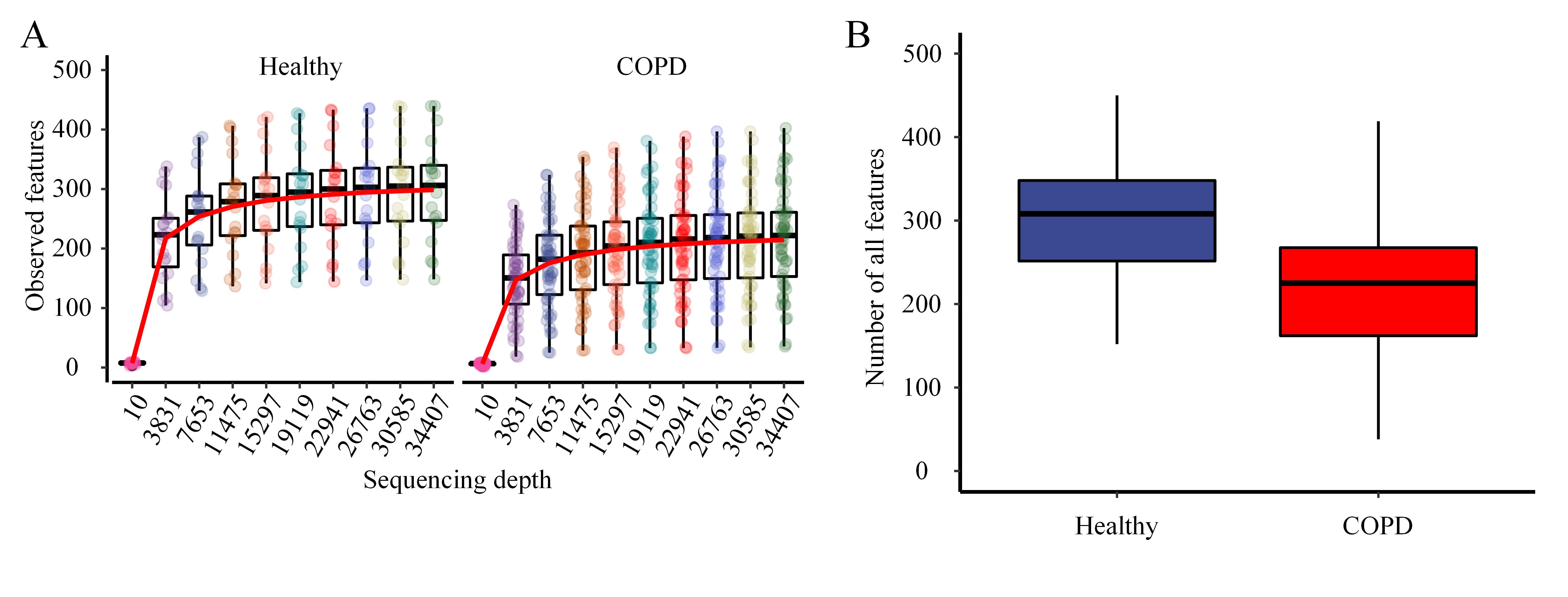


**Fig S1** The rarefaction analysis between the number of samples and the number of ASVs features. A) Sampling depth reaches the lowest frequency, ASVs features has reached the plateau. B) All observed ASVs features in different groups.


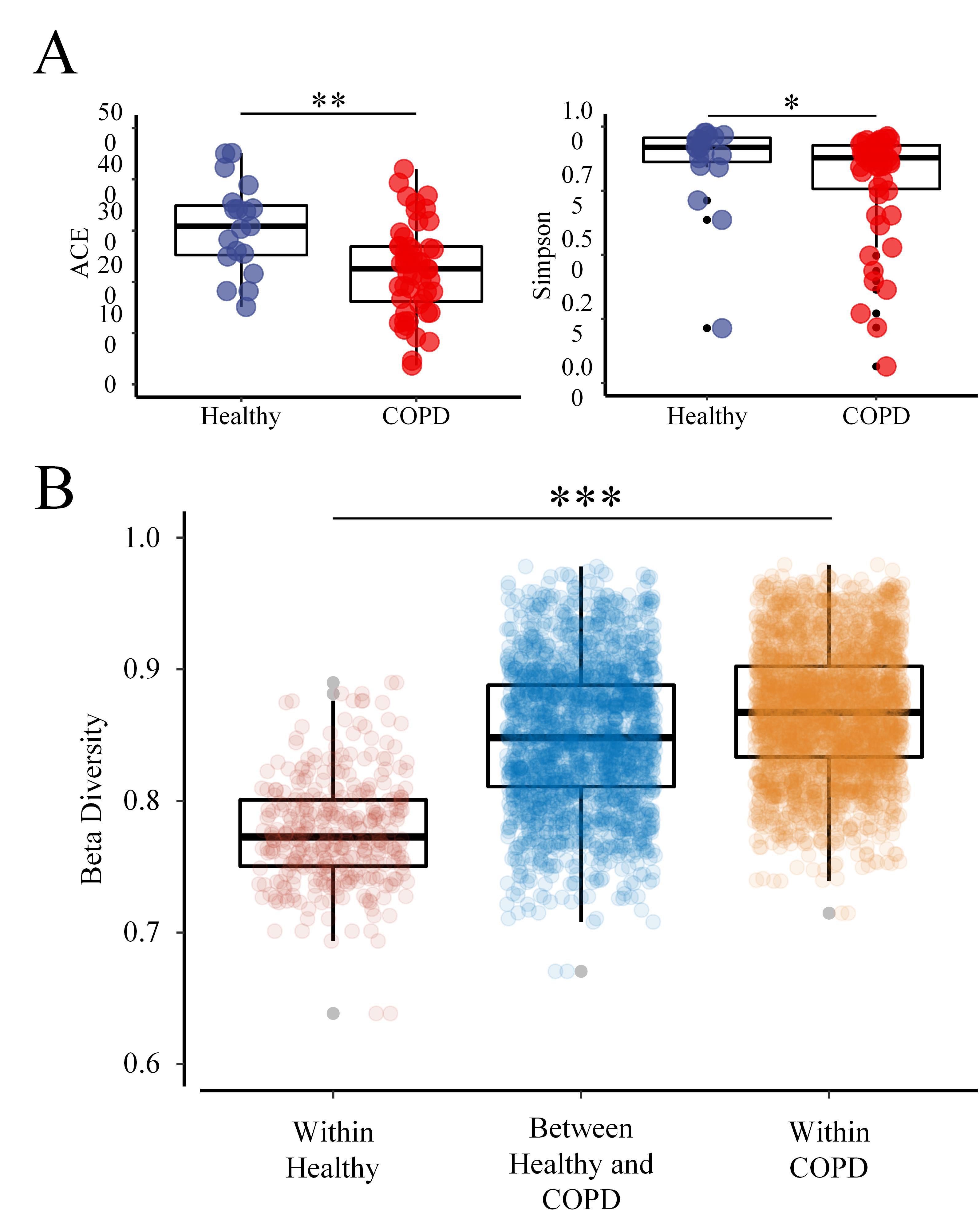


**Fig S2** Sputum microbial diversity of COPD patients was decreased. A) As estimated by ACE index, Simpson index, sputum microbial diversity was significantly decreased in COPD (n = 51) compared with that in the healthy controls (n = 19). B) Beta diversity analysis based on Jaccard distance and Wilcoxon test, distinction between healthy controls and COPD was statistically significant. *, *p* <0.05; **, *p*<0.01; *** *p*<0.001. COPD, Chronic obstructive pulmonary disease.


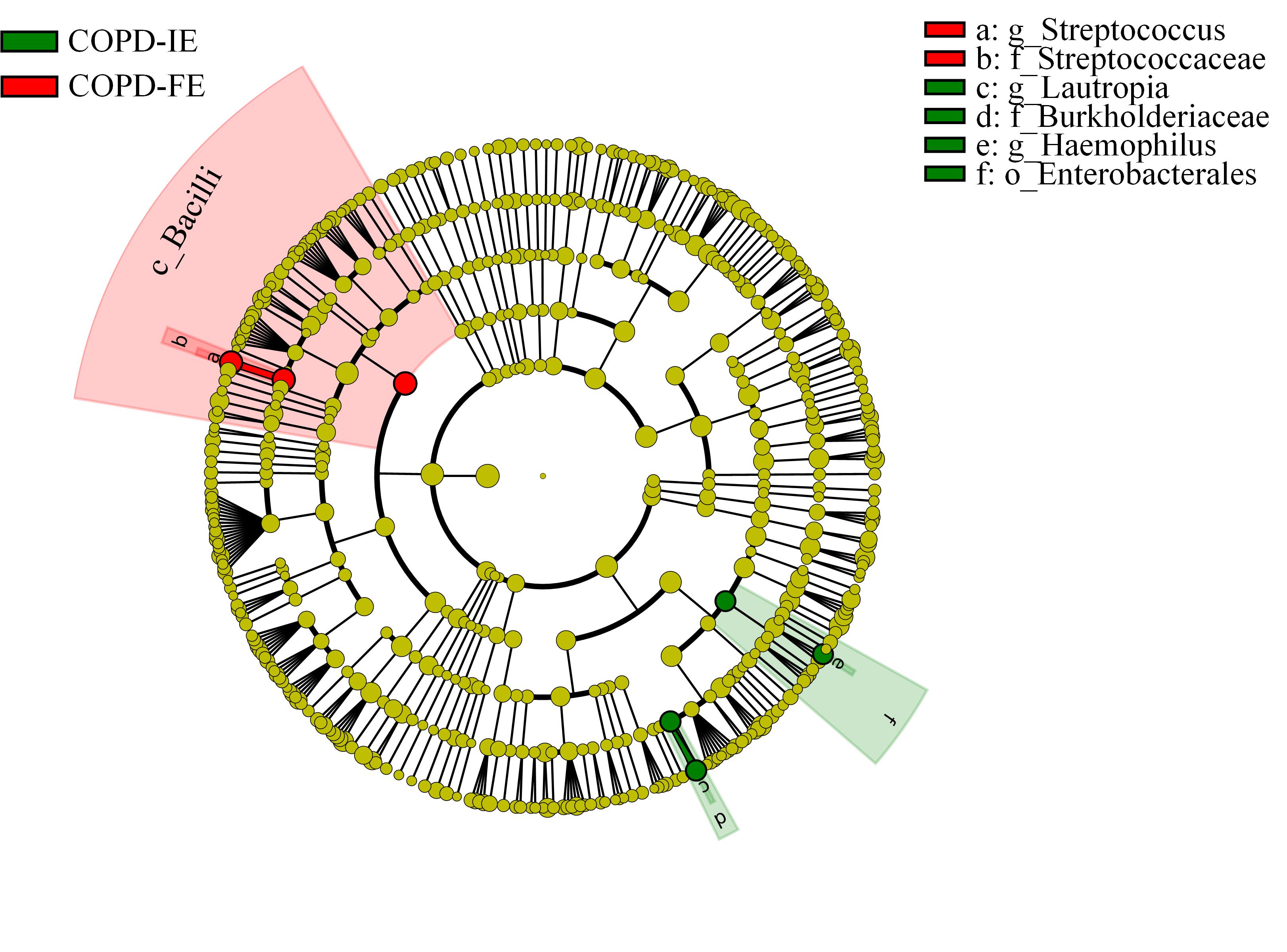


**Fig S3** Crucial bacteria of sputum microbiome related to COPD-FE. The phylogenetic profile of the specific bacterial taxa and the major bacteria associated with COPD-IE and COPD-FE.


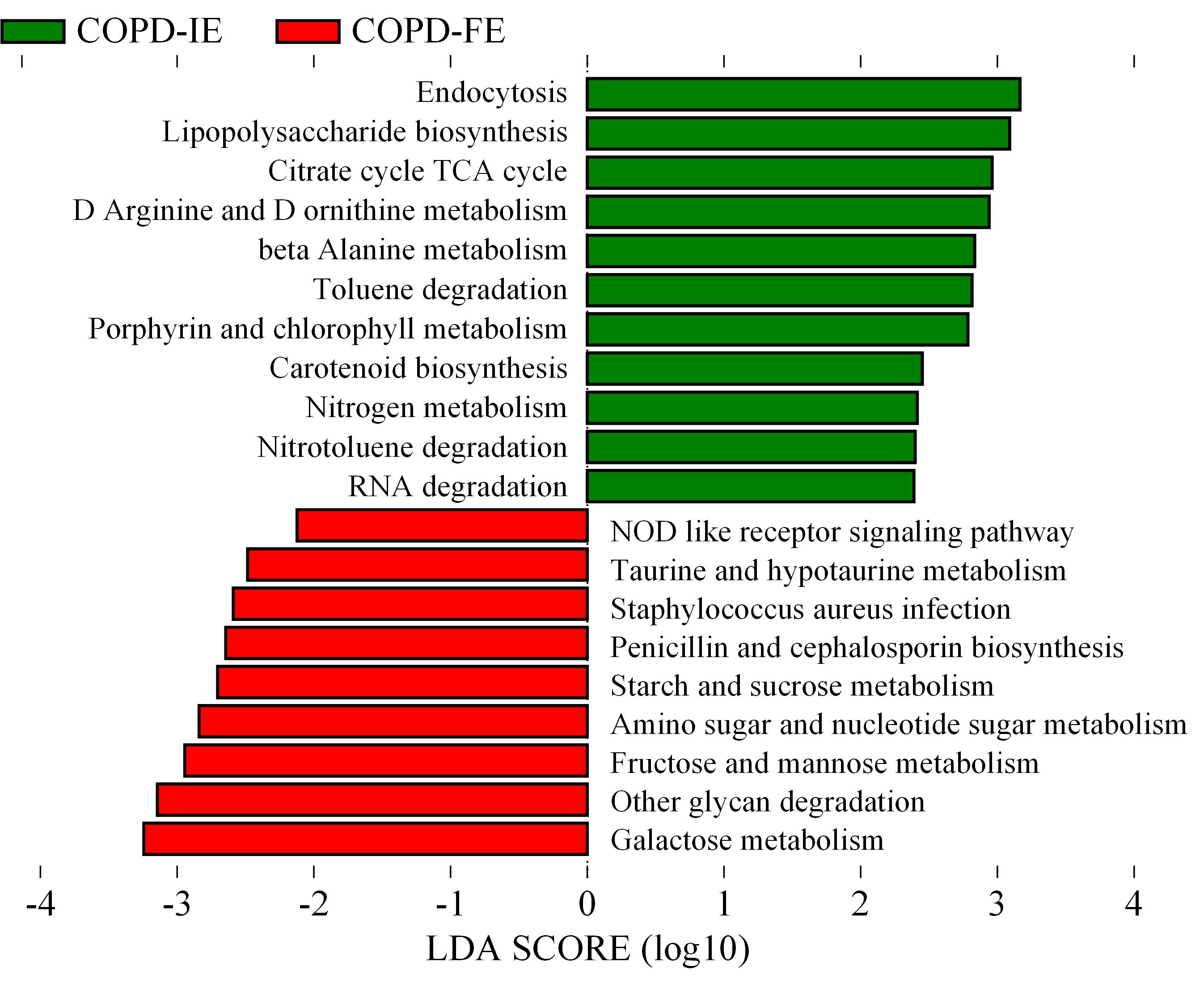


**Fig S4** Predicted functions related to frequent exacerbation phenotype. Based on the predicted KOs and KEGG pathways, nine predicted microbial functions were remarkably increased, while 11 functions were remarkably decreased in COPD-FE compared with COPD-IE. (LDA score (log10) = 2 is cutoff value): *p* < 0.05). COPD, Chronic obstructive pulmonary disease; COPD-IE, COPD with infrequent exacerbations; COPD-FE, COPD with frequent exacerbations ; LDA, linear discriminant analysis.
